# Diagnostic and Prognostic Factors in Acute Intestinal Ischemia: A Comparative, Retrospective, Single-Center Cohort Study

**DOI:** 10.3390/jcm15166157

**Published:** 2026-08-07

**Authors:** Musa Murat Çalışkan, Tahir Talat Yurttaş, Selim Doğan, Ufuk Oğuz İdiz, Kenan Büyükaşık, Taşkın Rakıcı, Mert Mahsuni Sevinç

**Affiliations:** 1 Department of General Surgery, Istanbul Training and Research Hospital, University of Health Sciences, 34098 Istanbul, Türkiye; 2 Department of Emergency Medicine, Istanbul Training and Research Hospital, University of Health Sciences, 34098 Istanbul, Türkiye; 3 Department of Radiology, Istanbul Training and Research Hospital, University of Health Sciences, 34098 Istanbul, Türkiye

**Keywords:** acute mesenteric ischemia, Mannheim peritonitis index, APACHE-II, neutrophil-to-lymphocyte ratio, arterial lactate, prognosis

## Abstract

**Background/Objectives:** Acute mesenteric ischemia (AMI) resembles non-vascular intestinal ischemia (NSII) and non-specific abdominal pain (NSAP) at presentation yet carries 50–80% mortality. We tested how well admission data separate these conditions and predict in-hospital mortality, the primary endpoint. **Methods:** We studied 197 consecutive adults (2015–2021): AMI (*n* = 61), NSII (*n* = 62), and NSAP controls (*n* = 74). Admission demographics, comorbidities, laboratory markers, the Mannheim Peritonitis Index (MPI), and APACHE-II were compared by ROC/DeLong analysis and multivariable logistic regression (STROBE). **Results:** AMI had more atrial fibrillation, thromboembolism, chronic kidney disease (CKD), and dyslipidemia (*p* ≤ 0.028) and higher in-hospital mortality than NSII (47.5% vs. 16.1%; *p* < 0.001). Inflammatory ratios, lactate, and albumin separated AMI from NSAP well (AUC up to 0.93) but from NSII modestly (lactate, 0.672). APACHE-II (AUC 0.861) and MPI (0.810) predicted in-hospital death and improved combined (0.897; DeLong *p* = 0.014). Independent predictors were MPI (adjusted odds ratio 1.14, 95% CI 1.06–1.23), CKD (3.72, 1.25–12.10), and arterial lactate (1.20, 1.03–1.45). **Conclusions:** A composite admission panel was internally consistent but hypothesis-generating, not a triage tool ready for clinical use. CT angiography remains essential, since these markers cannot separate vascular from non-vascular ischemia; prospective external validation is required.

## 1. Introduction

Acute mesenteric ischemia (AMI) is among the most lethal abdominal emergencies, with a reported mortality of 50–80% despite advances in cross-sectional imaging and revascularization [1]. Its early course is treacherous: pain is frequently out of proportion to unremarkable examination findings, and overt peritoneal signs emerge only once transmural necrosis has developed, so that survival is tightly coupled to the interval from symptom onset to revascularization or resection [2].

Because definitive imaging and the operating room are not always immediately at hand, the initial decision must rest on data available at admission—the physical examination, routine bloodwork, and arterial blood gas. Inflammatory and nutritional indices derived from the admission blood count—the neutrophil-to-lymphocyte ratio, the platelet-to-lymphocyte ratio, immature granulocytes, the prognostic nutritional index, and the recently described C-reactive-protein–albumin–lymphocyte (CALLY) index—together with metabolic markers such as arterial lactate, have shown diagnostic or prognostic promise [3]; yet, a systematic review and meta-analysis concluded that no single biomarker is sufficiently accurate on its own [4]. Successive efforts to identify a stand-alone diagnostic marker—D-dimer, intestinal fatty-acid-binding protein, and citrulline among them—have repeatedly fallen short of the specificity required for a rule-in test, and a recent international multicenter study likewise found that no routine laboratory value reliably identified arterial AMI [5,6]. Peritonitis-severity scores such as the Mannheim Peritonitis Index (MPI) and the Acute Physiology and Chronic Health Evaluation II (APACHE-II) provide a complementary, physiology-based estimate of risk [7]. These admission measures, however, have rarely been evaluated together and head-to-head in a surgical cohort that also includes a non-vascular (mechanical or strangulating) intestinal-ischemia comparator and a non-specific abdominal pain control group [8]—a frequent emergency-department presentation in which no specific surgical or organic cause is identified after clinical, laboratory, and imaging evaluation.

We therefore compared patients operated on for AMI with those operated on for non-vascular (extravascular) intestinal ischemia (NSII) and with non-specific abdominal pain (NSAP) controls, in order to determine how well admission demographics, comorbidities, biomarkers, and severity scores discriminate these conditions and predict early postoperative mortality. The primary endpoint was in-hospital mortality; the pre-specified secondary endpoints were 30-day and 90-day mortality and the diagnostic discrimination of AMI from NSII and from NSAP by admission markers and severity scores. We hypothesized that admission biomarkers would separate ischemia from non-specific pain but not vascular from non-vascular ischemia, and that physiology-based severity scores—rather than any single laboratory value—would carry the prognostic weight.

## 2. Materials and Methods

### 2.1. Study Design and Setting

This single-center, retrospective comparative cohort study screened all consecutive adults evaluated for acute abdominal pain at the Department of General Surgery, Istanbul Training and Research Hospital, between 1 January 2015 and 1 June 2021. It was approved by the hospital’s Clinical Research Ethics Committee (4 June 2021; approval no. 2862; reference 2011-KAEK-50) and conducted in accordance with the Declaration of Helsinki and institutional data-protection rules. Reporting follows the STROBE statement [9]; the prediction-model components additionally follow TRIPOD [10].

### 2.2. Patient Selection and Group Definitions

**Figure 1:**
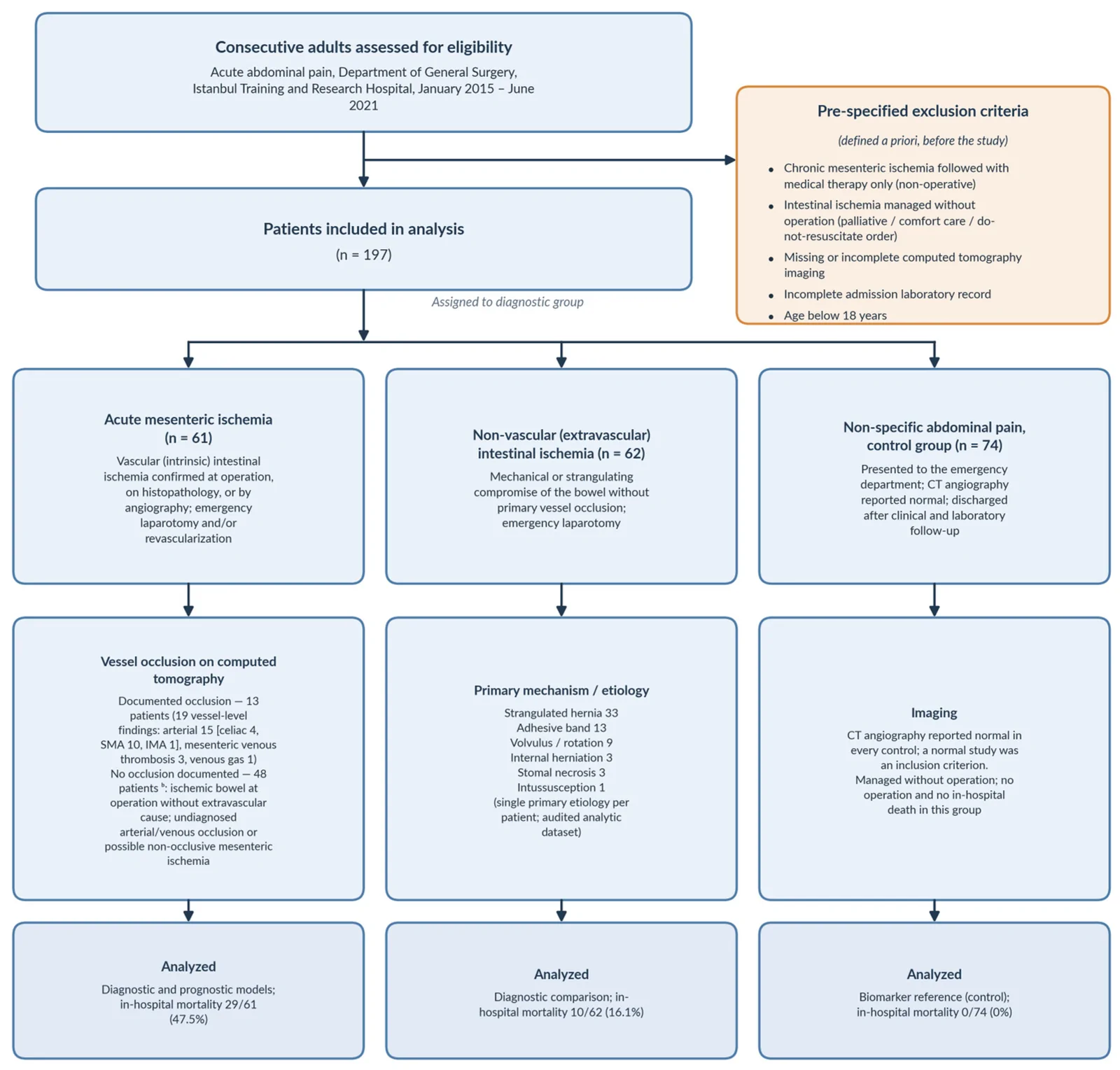
STROBE participant flow diagram. Exclusion criteria were pre-specified a priori, before study commencement; patients meeting any criterion were ineligible and were not enrolled. Because the audited analytic dataset contains only the 197 included patients, the number excluded under each criterion was not available for retrospective reconstruction. The Mannheim Peritonitis Index (MPI) and the Acute Physiology and Chronic Health Evaluation II (APACHE-II) score were computed only for operated patients (both ischemia groups); by design they are not applicable to the non-operative control group. ^b^ Among the 48 acute mesenteric ischemia patients with no vessel occlusion documented on CT (possible non-occlusive mesenteric ischemia), 35 of 48 (72.9%) showed at least one secondary ischemic CT sign (free fluid 27, air–fluid level 17, bowel-wall thickening 16, bowel dilation 16); 46 of 48 required bowel resection, and 23 of 48 died in hospital. The 2 patients not resected both survived but showed transmural ischemia (one with arterial lactate 6.73 mmol/L and MPI 14; the other with MPI 31 and purulent intra-abdominal exudate). AMI, acute mesenteric ischemia; NSII, non-vascular (extravascular) intestinal ischemia; NSAP, non-specific abdominal pain.

All 197 patients were assigned to one of three groups. The acute mesenteric ischemia (AMI) group (*n* = 61) had surgically and/or angiographically confirmed mesenteric vascular occlusion—arterial embolic or thrombotic, venous, or non-occlusive—and underwent emergency laparotomy and/or open or endovascular revascularization. Patients in whom computed tomography demonstrated no vessel occlusion but whose intra-operative luminal and mesenteric findings were ischemic in the absence of any extravascular pathology—together with those whose arterial or venous occlusion could not be fully characterized on imaging, or in whom non-occlusive mesenteric ischemia was suspected—were assigned to the AMI group on the basis of the combined intra-operative and histopathological assessment of arterial and venous status. The arterial, venous, and non-occlusive subtypes were analyzed as a single AMI group because they share the same final common pathway—transmural intestinal ischemia requiring emergency operation—and because the admission triage questions addressed here (ischemia versus non-ischemic pain, and vascular versus mechanical cause) precede subtype confirmation, which typically becomes available only on CT angiography or at laparotomy; the distribution of documented subtypes is reported in Section 3.1 and Figure 1.

In the non-vascular (extravascular) intestinal ischemia (NSII) group (*n* = 62), the bowel was compromised by a mechanical or strangulating cause rather than by primary vessel occlusion; audited primary etiologies were strangulated hernia (*n* = 33), adhesive band (*n* = 13), volvulus or rotation (*n* = 9), internal herniation (*n* = 3), stomal necrosis (*n* = 3), and intussusception (*n* = 1). Fifty-nine of these 62 cases (95.2%) reflected strangulating or closed-loop mechanical compromise, in which luminal or pedicle obstruction secondarily occludes the intramural microcirculation; the three stomal-necrosis cases (4.8%) instead represent a localized failure of stomal perfusion and were retained within NSII because they too produce intestinal ischemia without primary mesenteric-vessel occlusion. Group assignment reflected the intra-operative determination of intestinal ischemia rather than whether a resection was performed; bowel that was frankly ischemic at operation but recovered after relief of the mechanical cause (detorsion of a volvulus, division of an adhesive band, or reduction of a strangulated hernia) was salvaged without resection and retained in NSII, with viability confirmed directly or at a planned second-look laparotomy.

The non-specific abdominal pain (NSAP) control group (*n* = 74) comprised patients who presented to the emergency department with non-specific abdominal pain, underwent CT angiography that was reported as normal, and were discharged from the emergency department after clinical follow-up and admission laboratory results revealed no organic surgical cause. A normal CT angiogram was thus an explicit inclusion criterion for this group, which is why no positive structured CT finding was recorded in any control, and all 74 controls were managed entirely non-operatively. Follow-up for controls was confined to the index admission; the absence of structured post-discharge surveillance for a missed early diagnosis is acknowledged as a limitation (Section 4.4).

We included adults (≥18 years) with a complete admission laboratory record—defined as availability of all core prespecified variables (complete blood count, creatinine, urea, and arterial blood gas with lactate); markers ordered selectively (Section 2.6) were permitted to be missing. We excluded chronic mesenteric ischemia followed with medical therapy only (non-operative); patients with intestinal ischemia who did not undergo an operation—those managed palliatively along comfort-care or do-not-resuscitate pathways—so that both ischemia groups comprise operated patients exclusively; missing or incomplete CT imaging; and an incomplete admission laboratory record. The number of patients screened and excluded at each step, with reasons, is summarized in the STROBE flow diagram (Figure 1); the exact counts excluded before group assignment were not retained in the audited analytic dataset—which contains only the 197 included patients—and are to be completed from the institutional screening log.

### 2.3. Data Collection

The electronic medical records were independently reviewed, and any discrepancies were resolved by consensus. Variables comprised demographics (age, sex, weight, height); fourteen binary comorbidities (atrial fibrillation, prior thromboembolism, chronic kidney disease, dyslipidemia, hypertension, diabetes, heart failure, coronary artery disease, cerebrovascular disease, chronic obstructive pulmonary disease, hypercoagulable state, inflammatory bowel disease, active smoking, and COVID-19); admission examination (diffuse tenderness, guarding or rebound); complete blood count, routine biochemistry, arterial blood gas, and coagulation studies; contrast-enhanced CT angiography findings; intra-operative findings (ostomy formation, second-look laparotomy, length of bowel resected by anatomical segment; and intra-operative assessment of bowel viability—serosal appearance, peristalsis, mesenteric arterial pulsation, and bleeding from cut resection margins, supplemented by a planned second-look laparotomy when viability was uncertain); histopathology; postoperative intensive care unit admission; length of stay; and in-hospital, 30-day, and 90-day mortality.

### 2.4. Outcomes and Endpoints

The primary endpoint was in-hospital (index-admission) mortality, chosen because it is the most objective and least loss-prone outcome in this retrospective cohort and because, in fulminant mesenteric ischemia, death is the dominant hard outcome that early triage seeks to prevent. Thirty-day and 90-day mortality were secondary endpoints. Because calendar dates of death were not recorded, and every death within 90 days occurred during the index admission, in-hospital and 90-day mortality coincide, while the 30-day estimate is marginally lower because three AMI deaths occurred in hospital beyond post-admission day 30 (Section 3.4); the 90-day figure is therefore effectively an in-hospital endpoint, and in-hospital mortality is reported as the reference outcome throughout. Non-fatal complications (e.g., anastomotic leak, wound infection, unplanned re-operation) were not adopted as endpoints because they were inconsistently documented across the six-year period and were largely inapplicable to the non-operative NSAP group; operative morbidity is instead described through length of stay, intensive-care admission, resection extent, and second-look laparotomy.

### 2.5. Definitions

AMI was defined by intra-operative or angiographic confirmation of celiac, superior, or inferior mesenteric arterial occlusion, mesenteric venous thrombosis, or porto-mesenteric venous gas with corresponding bowel ischemia [1]. NSII was defined by intra-operative bowel ischemia attributable to extravascular mechanical compromise without primary mesenteric-vessel occlusion. Resection extent was recorded by anatomical segment using standard surgical distances. On imaging, bowel dilatation was defined as >2.5 cm (small bowel), >6 cm (colon), and >9 cm (caecum), and wall thickening as >2 mm.

### 2.6. Severity Scoring

The Mannheim Peritonitis Index (MPI) was determined for every patient with intestinal ischemia using the original ten-component instrument [11]: age > 50 years (5 points), female sex (5), organ failure (7), malignancy (4), preoperative sepsis > 24 h (4), non-colonic source (4), diffuse generalized peritonitis (6), and intra-abdominal exudate scored as clear (0), purulent (6), or feculent (12); organ failure followed the original MPI criteria. The APACHE-II score [12] was calculated for the 76 intensive-care-admitted patients with a complete first-24 h physiological dataset (of 83 postoperative ICU admissions), using the worst physiological values within the first 24 h; in the remaining seven, some component tests had not been requested, so a valid APACHE-II score could not be computed, and it is absent from the intensive-care records. Both indices remain validated severity tools for non-traumatic abdominal emergencies [7].

### 2.7. Laboratory Variables and Pre-Specified Exclusions

Admission inflammatory ratios were derived from the routine complete blood count: the neutrophil-to-lymphocyte ratio (NLR), the platelet-to-lymphocyte ratio (PLR), and the immature-granulocyte percentage; the prognostic nutritional index was derived from albumin and lymphocyte count. Markers measured in fewer than 30% of the cohort overall (D-dimer, 19/197; procalcitonin, 23/197; C-reactive protein, 30/197; lactate dehydrogenase, 41/197) were excluded from the principal between-group comparisons because of insufficient power and near-absent control-group sampling, and are referenced in the Discussion only for literature comparison. For markers retained in the between-group comparisons, the number of patients contributing to each analysis varied with test availability and is reported per group in the corresponding table footnote (for example, arterial lactate was available in 54/34/18 and albumin in 52/51/23 AMI/NSII/NSAP patients).

### 2.8. Data-Integrity Audit and Reproducibility

This study derives from a source medical-specialty thesis [13], in which normality was assessed on pooled model residuals (the “residual” rule) and the analyses were performed with IBM SPSS Statistics 23 (IBM Corp., Armonk, NY, USA), jamovi 1.6.23 (The jamovi project, Sydney, Australia), and JASP 0.14.1.0 (JASP Team, University of Amsterdam, Amsterdam, The Netherlands). The raw dataset subsequently underwent a documented internal audit limited to record-by-record verification and correction of data-entry errors; no patients, variables, or measurements were added or removed. The complete analysis is openly published on Zenodo [14] and can be inspected one-to-one; the repository provides an exact reproduction of the original thesis results (unaudited data, residual rule), which was re-run in R 4.6.0 so that the thesis figures are reproduced exactly, together with the definitive analysis used here, which applies a traditional SPSS Explore-style per-group normality selection (Shapiro–Wilk evaluated within each diagnostic group) to the audited data. All results reported in this article were generated from the audited per-group analysis, which serves as the single analytic reference; the analysis code, statistical outputs, and per-project methodological details are available in the repository [14].

### 2.9. Statistical Analysis

Analyses were performed in R 4.6.0 (R Foundation for Statistical Computing, Vienna, Austria) using the packages pROC 1.19.0.1, PMCMRplus 1.9.12, car 3.1-5, rstatix 0.7.3, and survival 3.8-6, the last being one of the recommended packages distributed with R 4.6.0. The complete analysis code, the per-script sessionInfo records, and the corresponding renv lockfile are openly available [14]. Normality was assessed by the Shapiro–Wilk test with histogram and quantile–quantile inspection, applying a per-group (SPSS Explore-style) rule in which the Shapiro–Wilk test was evaluated separately within each diagnostic group; homogeneity of variance was assessed by the Levene test. Because most continuous variables were non-normally distributed, three-group comparisons used the Kruskal–Wallis test with Dwass–Steel–Critchlow–Fligner post hoc analysis; where every group was normally distributed, one-way analysis of variance with the Tukey honestly significant difference post hoc test was used when variances were homogeneous, and Welch analysis of variance with the Games–Howell post hoc test when they were not. The corresponding two-group tests were the Mann–Whitney U test and the Student or Welch *t*-test, selected by the same rule. Categorical variables were compared by the Pearson chi-square test, or the Fisher exact test when any expected count was below 5. Discrimination of AMI from NSII (and from NSAP) was assessed by ROC analysis, reporting the area under the curve (AUC) with DeLong 95% confidence intervals and Youden-derived cut-offs, with paired AUCs compared by the DeLong test. Independent predictors were derived from prespecified multivariable binary logistic regression (Enter method), expressed as adjusted odds ratios with profile-likelihood 95% confidence intervals; the AMI-risk model included atrial fibrillation, prior thromboembolism, chronic kidney disease, NLR, and arterial lactate (dyslipidemia was excluded for perfect separation), and the mortality models comprised age, MPI, NLR, arterial lactate, atrial fibrillation, and chronic kidney disease. Internal validity was assessed by Harrell’s bootstrap optimism correction of the AUC (B = 1000) and by the Hosmer–Lemeshow goodness-of-fit test over deciles of predicted risk, both implemented in base R; net benefit by decision-curve analysis; and in-hospital survival (the primary endpoint) by the Kaplan–Meier method with the log-rank test. Multiplicity across the laboratory panel was controlled by the Benjamini–Hochberg false-discovery-rate procedure. A two-sided *p* < 0.05 was considered significant.

### 2.10. Ethics and Data Availability

The Clinical Research Ethics Committee of Istanbul Training and Research Hospital approved the study (4 June 2021; approval no. 2862; reference 2011-KAEK-50). Because all patients had signed the institutional general consent authorizing secondary scientific use of de-identified clinical data, no separate study-specific consent was required for this retrospective analysis; the working dataset contains no names, identifiers, or admission dates. The complete R analysis code, statistical outputs, figures, and Supplementary Material Tables are openly available in the Zenodo repository [14]; the de-identified individual-patient dataset is available from the corresponding author on reasonable request, in accordance with the original institutional protocol.

## 3. Results

### 3.1. Cohort and Baseline Characteristics

Of 197 patients, 61 had acute mesenteric ischemia (AMI), 62 had non-vascular (extravascular) intestinal ischemia (NSII), and 74 formed the non-specific abdominal pain (NSAP) control group (Figure 1). Age differed across the three groups (AMI 63.9 ± 15.9, NSII 63.2 ± 17.4, NSAP 56.0 ± 12.9 years; *p* < 0.001, Kruskal–Wallis), driven by the younger control group; the two operative groups were of similar age (*p* = 0.94). Sex distribution was comparable (female 60.7%, 50.0%, and 55.4%; *p* = 0.49).

Across the full three-group comparison (Table 1), cardiovascular and renal comorbidity followed a gradient from AMI through NSII to NSAP; chronic kidney disease (36.1% vs. 14.5% vs. 0%; *p* < 0.001), hypercoagulable state (27.9% vs. 19.4% vs. 2.7%; *p* < 0.001), atrial fibrillation (31.1% vs. 14.5% vs. 10.8%; *p* = 0.006), heart failure (19.7% vs. 11.3% vs. 2.7%; *p* = 0.006), cerebrovascular disease (14.8% vs. 6.5% vs. 1.4%; *p* = 0.010), and prior thromboembolism (13.1% vs. 1.6% vs. 1.4%; *p* = 0.003) were each most frequent in AMI. Restricting the analysis to the two operative groups (Table 2), atrial fibrillation (31.1% vs. 14.5%; *p* = 0.028), prior thromboembolism (13.1% vs. 1.6%; *p* = 0.017), chronic kidney disease (36.1% vs. 14.5%; *p* = 0.006), and dyslipidemia (8.2% vs. 0.0%; *p* = 0.028) were significantly more common in AMI than in NSII, whereas the remaining comorbidities were similar. Dyslipidemia did not reach significance in the three-group comparison (*p* = 0.051), where it was most frequent in the NSAP control group (9.5%).

Within the AMI group, a vessel-level occlusion was documented on computed tomography in 13 patients; these 13 accounted for 19 vessel-level findings (arterial in 15 [celiac four, superior mesenteric artery 10, inferior mesenteric artery one], mesenteric venous thrombosis in three, and porto-mesenteric venous gas in one), with several patients showing multi-vessel involvement, whereas in the remaining 48 no vessel occlusion was demonstrated on cross-sectional imaging, and the diagnosis was established at laparotomy (consistent with undiagnosed arterial or venous occlusion or possible non-occlusive mesenteric ischemia). Subtype-specific 90-day mortality was 60.0% (6/10) among patients with a documented arterial occlusion and 47.9% (23/48) in the no-occlusion (possible non-occlusive) subgroup, with no deaths among the three venous cases. Mortality did not differ across the arterial, venous, and non-occlusive subtypes (Fisher exact *p* = 0.29), and it was similar in the no-occlusion and documented-occlusion subgroups (23/48 vs. 6/13; *p* = 1.00), supporting their analysis as a single AMI group (Section 2.2). Among these 48 no-occlusion patients, 35 (72.9%) exhibited at least one secondary ischemic CT sign (free fluid in 27, air–fluid level in 17, bowel-wall thickening in 16, and bowel dilation in 16) and 46 (95.8%) required bowel resection, supporting a true ischemic process rather than viable-bowel recovery. In NSII, 59 of 62 mechanisms (95.2%) were strangulating or closed-loop, and three (4.8%) were stomal necrosis; bowel resection was performed in 38 of 62 (61.3%), whereas in 24 (38.7%), frankly ischemic bowel recovered after relief of the mechanical cause and was salvaged without resection (in-hospital mortality 2/24 [8.3%] after salvage and 8/38 [21.1%] after resection; Fisher exact *p* = 0.29). Contrast-enhanced CT was performed in 54/61 AMI and 55/62 NSII patients, with dedicated CT angiography in 10/61 and 2/62 and diagnostic catheter angiography in 20/61 AMI patients; the remaining operative diagnoses were made at laparotomy. In NSAP, a normal CT angiogram was an inclusion criterion, so every control underwent cross-sectional imaging that was reported as normal; because no positive finding was recorded in any of them, the structured imaging variables are zero throughout the control group in the deposited dataset. Within the operative groups, by contrast, CT angiography was applied selectively—to plan revascularization or when the diagnosis was uncertain—rather than uniformly, a point relevant to interpreting between-group imaging differences.

### 3.2. Admission Laboratory Findings and Diagnostic Discrimination

**Figure 2:**
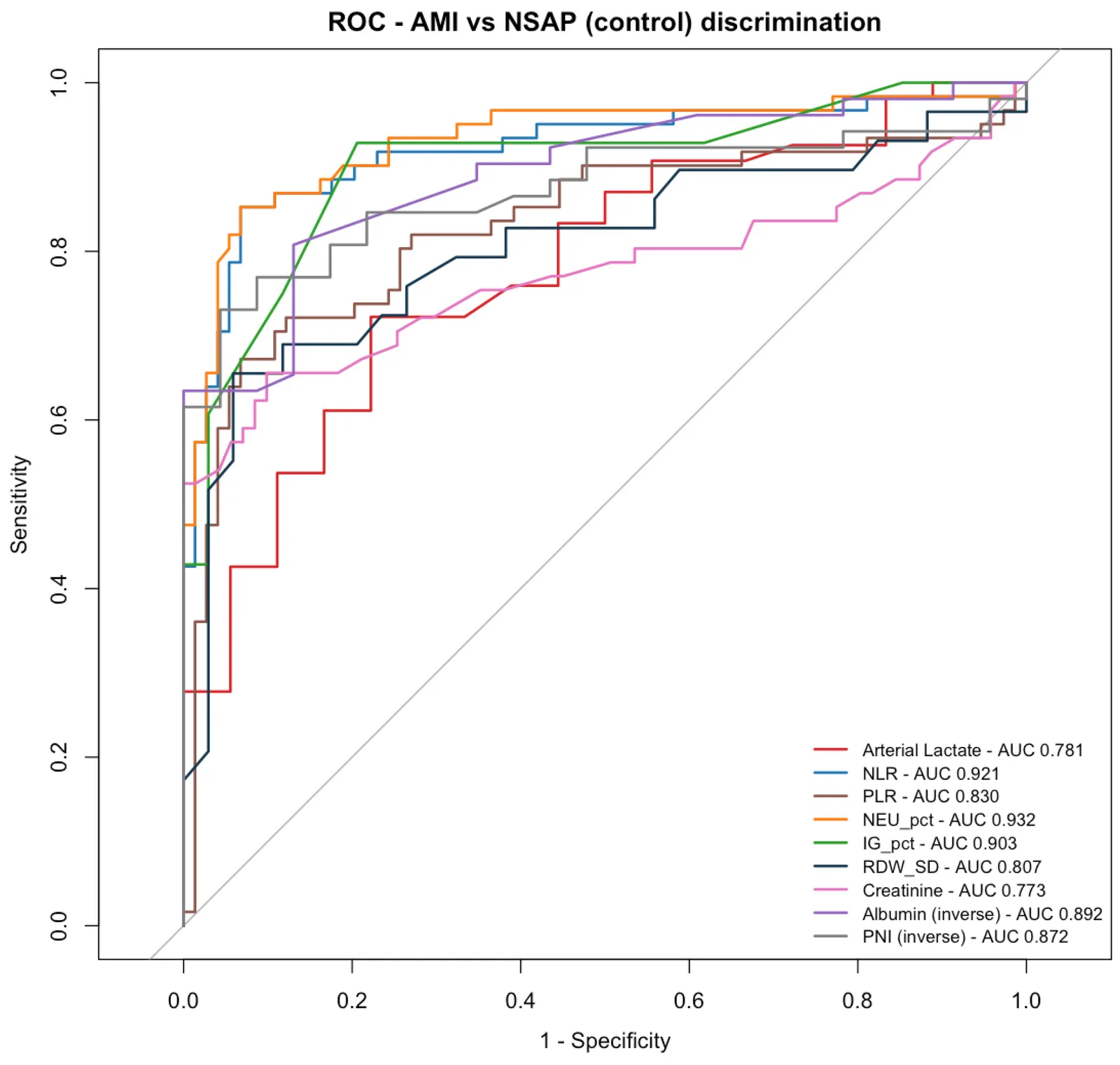
ROC curves of admission biomarkers discriminating AMI from NSAP controls.

**Figure 3:**
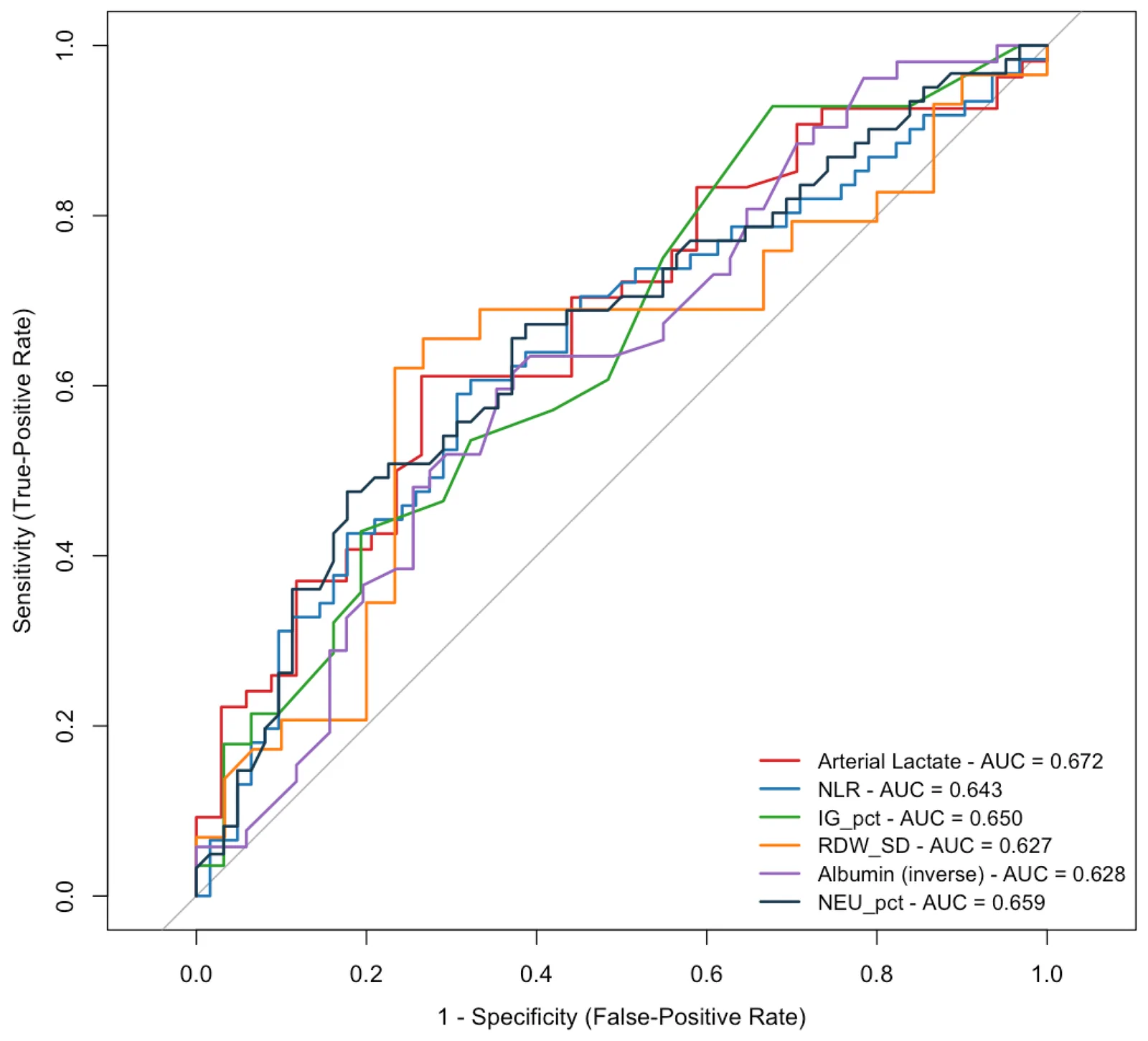
ROC curves of admission biomarkers discriminating AMI from NSII.

Across the three groups, 36 of 57 laboratory markers remained significantly different after Benjamini–Hochberg correction, with the inflammatory and nutritional indices showing the largest separations (Table 3). On ROC analysis, admission markers distinguished AMI from the NSAP control group with high accuracy—neutrophil percentage (area under the curve [AUC] 0.932), neutrophil-to-lymphocyte ratio (NLR; 0.921), immature granulocytes (0.903), albumin (0.892), and the prognostic nutritional index (PNI; 0.872) (Table 4, Panel A; Figure 2)—but separated the two operative groups (AMI vs. NSII) only modestly, the best single marker being arterial lactate (AUC 0.672, 95% CI 0.56–0.79) (Table 4, Panel B; Figure 3).

### 3.3. Severity, Operative, and Outcome Characteristics

The Mannheim Peritonitis Index (MPI) was higher in AMI than NSII (23.0 ± 9.3 vs. 17.5 ± 7.4; *p* < 0.001), while body mass index was similar (*p* = 0.27). AMI patients had a longer hospital stay (median 10 vs. 7 days; *p* = 0.003), greater small-bowel resection length (median 87.5 vs. 30 cm; *p* < 0.001), and more frequent postoperative intensive-care admission (86.9% vs. 48.4%; *p* < 0.001), together with more advanced peritoneal contamination. The primary endpoint, in-hospital mortality, was almost threefold higher in AMI than NSII (47.5% vs. 16.1%; *p* < 0.001); the secondary 30-day (42.6% vs. 16.1%; *p* = 0.001) and 90-day (47.5% vs. 16.1%; *p* < 0.001) estimates diverged only slightly; in-hospital and 90-day mortality coincided (both 47.5%) because every death within 90 days occurred during the index admission, whereas the 30-day estimate was marginally lower (42.6%) because three AMI patients died in hospital beyond post-admission day 30 (Table 5).

### 3.4. Predictors of Mortality

**Figure 4:**
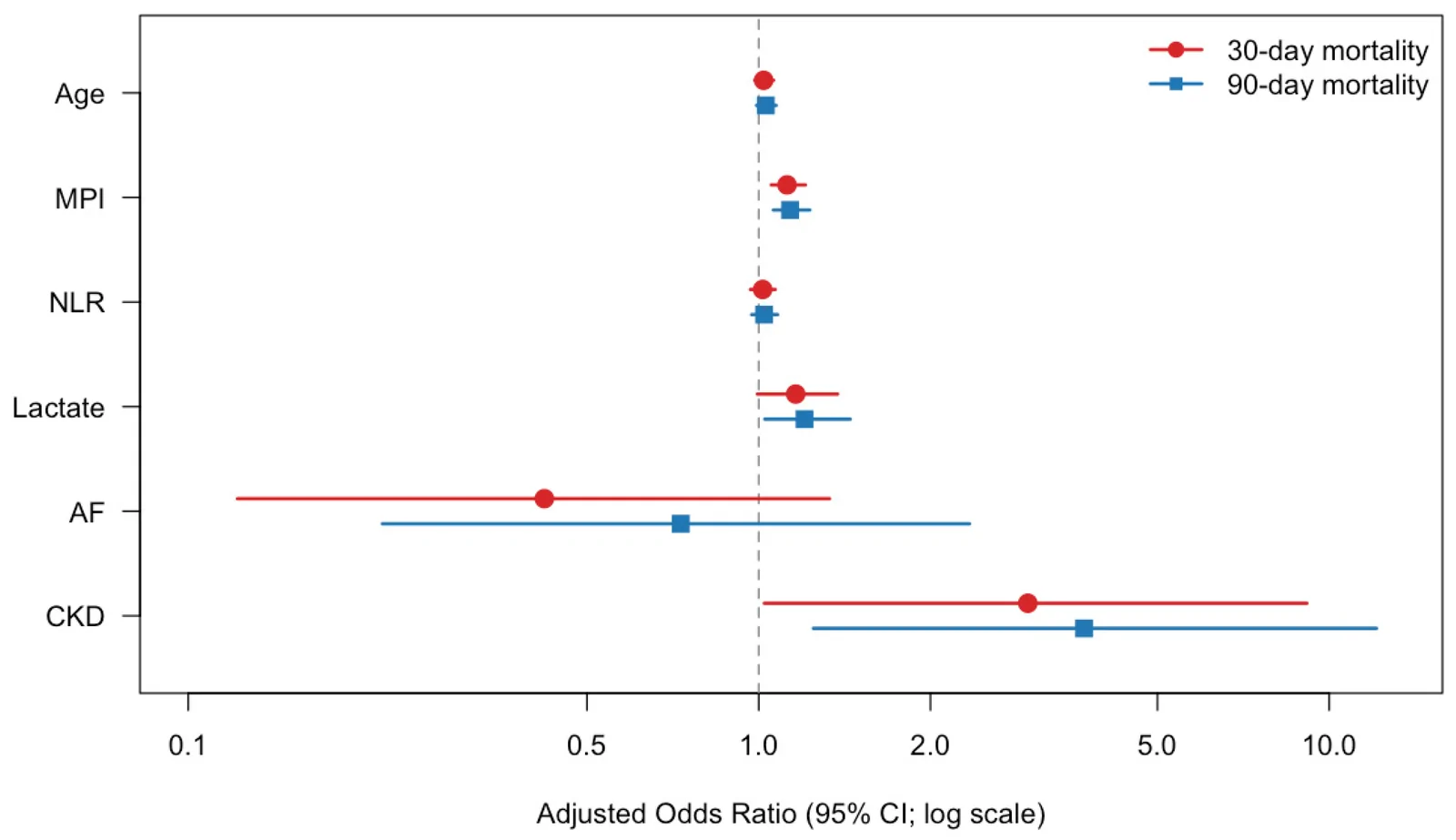
Adjusted odds ratios (forest plot) for 30-day and 90-day mortality.

**Figure 5:**
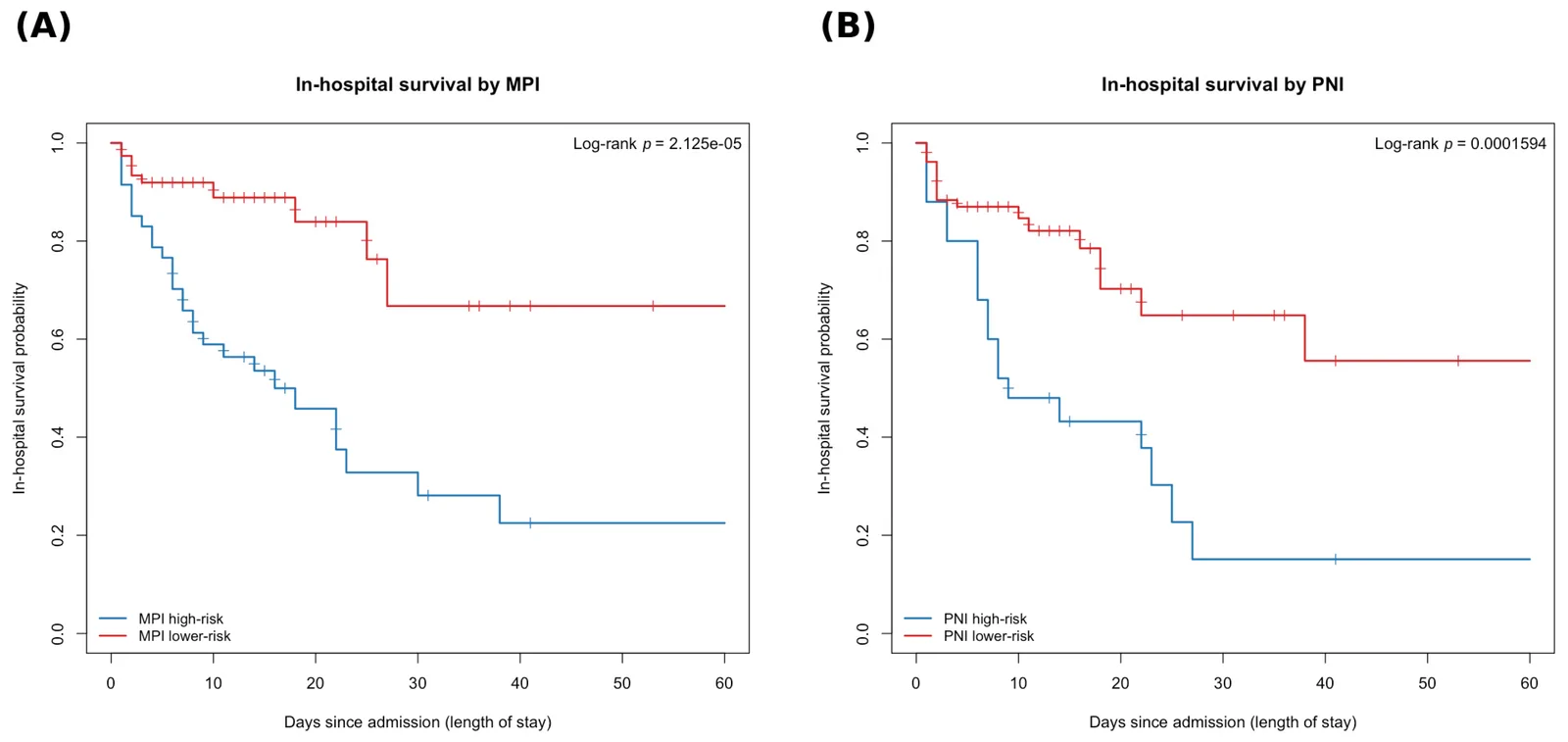
Kaplan–Meier in-hospital survival stratified by (**A**) MPI > 21.5 and (**B**) PNI < 31.1.

Within the operative cohort (AMI + NSII, *n* = 123), patients who died had higher MPI (26.7 ± 8.1 vs. 17.2 ± 7.4; *p* < 0.001) and APACHE-II scores (29.8 ± 10.6 vs. 15.4 ± 7.3; *p* < 0.001) and were older (69.0 vs. 61.0 years; *p* = 0.018). For in-hospital death (the primary endpoint), APACHE-II (AUC 0.861) and MPI (0.810) showed the strongest single-marker discrimination, followed by PNI (0.755), arterial lactate (0.694), and NLR (0.664); combining MPI and APACHE-II improved discrimination over MPI alone (AUC 0.897; DeLong *p* = 0.014) but not over APACHE-II alone (DeLong *p* = 0.257) (Table 6). On multivariable logistic regression, independent predictors of 90-day mortality (equivalent to in-hospital mortality, as all deaths occurred in hospital) were MPI (adjusted odds ratio [aOR] 1.14 per point, 95% CI 1.06–1.23; *p* < 0.001), chronic kidney disease (3.72, 1.25–12.10; *p* = 0.022), and arterial lactate (1.20, 1.03–1.45; *p* = 0.031); for 30-day mortality, MPI (1.12; *p* = 0.001) and chronic kidney disease (2.96; *p* = 0.0496) were independent (Table 7; Figure 4). After bootstrap correction, optimism-corrected discrimination was 0.69 for the AMI-risk model (Hosmer–Lemeshow *p* = 0.79) and 0.73 for the 30-day mortality model; the latter had a limited events-per-variable ratio (5.8) and imperfect calibration (Hosmer–Lemeshow *p* = 0.009) and is reported as exploratory; the corresponding internal-validation calibration and decision-curve analyses are provided in the Zenodo repository [14]. On Kaplan–Meier analysis, an MPI > 21.5 and a PNI < 31.1 each identified worse in-hospital survival (log-rank *p* < 0.001 for both) (Figure 5). The mortality models were fitted in the 88 operated patients with complete data for every model covariate (35 events at 30 days and 38 at 90 days). Because of this limited sample size and the correspondingly low events-per-variable ratio, the regression coefficients should be interpreted with caution; the adjusted odds ratios are exploratory, their confidence intervals are wide—most conspicuously for chronic kidney disease (1.25–12.10)—and they are best read as directional signals rather than as precise effect estimates.

## 4. Discussion

We compared three groups of patients who often look alike on arrival—AMI, non-vascular (extravascular) intestinal ischemia (NSII), and non-specific abdominal pain (NSAP)—using only the information available at admission. The pattern was clear. Routine inflammatory and nutritional indices told transmural ischemia apart from simple abdominal pain very well but did a poor job of separating the vascular and non-vascular forms. Death after operation was common and fell mostly on the AMI group (in-hospital 47.5% vs. 16.1%). What predicted it was not any single laboratory value but the physiology-based severity scores; APACHE-II and the Mannheim Peritonitis Index (MPI) gave the best single-marker discrimination (AUC 0.861 and 0.810) and did better together (0.897; DeLong *p* = 0.014). On regression, only MPI, chronic kidney disease, and arterial lactate held up as independent predictors of early death. Read against the current literature, these are largely confirmatory rather than novel findings, and we frame them accordingly below.

### 4.1. Discriminating Acute Ischemia from Non-Specific Abdominal Pain

Admission neutrophil percentage, the neutrophil-to-lymphocyte ratio (NLR), the immature-granulocyte fraction, albumin, and the prognostic nutritional index (PNI) each separated AMI from non-specific abdominal pain with areas under the curve of 0.87 to 0.93. Other recent surgical series report the same. Bolat and colleagues found diagnostic AUCs of 0.89, 0.86, and 0.81 for the systemic immune-inflammation index, NLR, and PNI in a comparison of AMI with undifferentiated abdominal pain [15], and the CALLY index has shown a weaker mortality signal (AUC 0.64) [3]. Our values therefore confirm, rather than extend, this body of work; they reproduce the magnitude of discrimination already reported and add a matched non-vascular comparator against which the same markers fail (Section 4.2). This fits the physiology: transmural ischemia provokes a brisk neutrophil response and a sharp drop in albumin, so indices built from these two move early. The caveat is that no single index is good enough on its own. A systematic review and meta-analysis reached exactly that conclusion [4], and a subsequent systematic review of fourteen candidate biomarkers similarly found none accurate enough to stand alone [6]. Our own figures need the same caution—several of the highest AUCs (immature granulocytes, RDW, albumin, PNI) came from smaller denominators, because those tests were ordered selectively in the control group. Selective ordering of this kind inflates apparent accuracy, so these values are best read as upper bounds.

A further caveat concerns the timing of presentation. Inflammatory indices rise over hours, so a patient reaching hospital very early in the ischemic process may still show a near-normal NLR, whereas the same marker is grossly abnormal once necrosis is established. Because AMI classically presents early with pain out of proportion to examination, admission inflammatory markers may understate severity in hyperacute cases and overstate it in late presenters; the single admission time-point captured here cannot model this kinetic, which may partly explain why tissue-damage markers grade severity better than they localize cause [6]. Population data reinforce the vascular-risk framing of the disease: in a prospective cohort of 28,098 individuals, smoking, alcohol, and physical inactivity independently predicted incident AMI [16], echoing the comorbidity gradient we observed across the three groups.

### 4.2. The Diagnostic Ceiling Between Vascular and Non-Vascular Ischemia

The harder question—separating arterial or venous AMI from a mechanical or strangulating cause—exposed the limits of admission bloodwork. Arterial lactate was the best single marker here, and it reached only 0.672; NLR and neutrophil percentage did no better than 0.64 to 0.66. The reason is mechanistic. Both conditions end in the same place, transmural injury and necrosis, so a marker that reports tissue damage cannot say whether the cause was vascular or mechanical. The signals that flag AMI rise in mechanical compromise too; NLR independently predicted bowel ischemia in non-strangulated adhesive obstruction (odds ratio 5.9) [17], and lactate, neutrophil count, and NLR tracked the need for resection in incarcerated hernia [18]. An international multicenter study reached the same ceiling from the arterial side, finding that no routine laboratory value reliably identified arterial AMI even against a mixed non-AMI comparator [5]. We therefore read these indices as measures of ischemic severity rather than of cause. The distinction that drives operative strategy—revascularization versus mechanical relief—still rests on CT angiography, which current guidelines place at the center of the work-up [1,2].

### 4.3. Prognostication: Lactate, Severity Scores, and a Composite Panel

The distinction between diagnostic and prognostic utility is worth stating plainly because the two are easily conflated. Lactate is a good example of a marker that is prognostically useful but diagnostically weak. It separated AMI from controls only moderately (0.781) and from non-vascular ischemia poorly (0.672), yet it remained an independent predictor of 90-day death (adjusted odds ratio 1.20 per mmol/L) [19]. The same dissociation appears elsewhere: in a multicenter study lactate failed as a diagnostic test for AMI (AUC ≈ 0.57–0.60) but still predicted intensive-care mortality whatever the diagnosis [20], and after aortic dissection severe lactatemia marked the patients for whom laparotomy was futile [21]. Consistent with this, dynamic lactate indices are outperformed by APACHE-II and SOFA for mortality in sepsis [22]. Lactate grades global hypoperfusion; it does not localize it.

The severity scores carried the prognostic load. APACHE-II and MPI each discriminated in-hospital death well (0.861 and 0.810), and combining them improved on MPI alone (0.897; DeLong *p* = 0.014) though not on APACHE-II alone (*p* = 0.257), so the gain is attributable chiefly to APACHE-II; this is in line with the prognostic value of MPI and APACHE-II across non-traumatic abdominal emergencies [7]. MPI was an independent predictor at both 30 and 90 days (adjusted odds ratio 1.12 and 1.14 per point). Kaplan–Meier stratification was equally telling: an MPI above 21.5 or a PNI below 31.1 marked clearly worse in-hospital survival (log-rank *p* < 0.001 for each), which suggests that nutritional depletion measured at admission adds prognostic information beyond peritoneal contamination. Chronic kidney disease was the strongest categorical predictor of death (adjusted odds ratio 3.72 at 90 days). Recent mortality models point the same way, with creatinine and renal dysfunction emerging as independent determinants of outcome [23,24,25]; renal impairment likely flags both the atherosclerotic burden behind mesenteric occlusion and the thin physiological reserve that decides whether a patient survives a major resection. Readily available inflammatory and metabolic biomarkers refine risk in other critically ill surgical populations [26], and stratification models built on such markers can rival SOFA and APACHE-II for in-hospital mortality [27]—supporting a composite-panel approach while underscoring that no single index suffices. The implication our data can suggest, but cannot establish, is a composite admission panel rather than any single number: inflammatory ratios to raise suspicion, lactate to grade severity, and MPI with APACHE-II to quantify risk. Decision-curve analysis was performed in the development data without optimism correction, and its result is correspondingly modest: net benefit exceeded both the treat-all and the treat-none strategy only at threshold probabilities above approximately 0.20 for the 30-day mortality model, whereas at a threshold of 0.10 the model did not outperform treating every patient, and the AMI-risk model likewise gained an advantage only at the highest thresholds examined. This pattern is worth stating plainly because the clinically relevant region for a lethal, time-critical diagnosis is the low-threshold region, in which a surgeon accepts many false positives in order not to miss transmural ischemia—precisely where the models conferred no advantage. The 30-day model also calibrated imperfectly (Hosmer–Lemeshow *p* = 0.009); these curves are therefore reported as an internal description of the present cohort rather than as evidence of clinical utility. A recent nomogram built on age, heart failure, leukocytosis, and creatinine reached similar discrimination (bootstrap AUC 0.85) in a separate AMI cohort [24,28].

These severity-score results must, however, be read against the way APACHE-II was obtained. The score was computed only in the 76 patients admitted to intensive care with a complete first-24 h physiological dataset, that is, in the sickest and most physiologically deranged segment of the operative cohort—precisely the stratum in which deaths are concentrated. Restricting the analysis to this subset introduces a selection bias that plausibly inflates the apparent discrimination of APACHE-II, both because the case mix is enriched for events and because the decision to admit a patient to intensive care is itself a clinical judgement of severity and is therefore correlated with the predictor and the outcome alike. The internal comparison in Table 6 makes the point directly: within the same ICU-scored subset the MPI area under the curve falls from 0.810 to 0.772, which shows that the composition of the subset, and not only the score itself, shapes the estimate. The APACHE-II value of 0.861 and the combined MPI + APACHE-II value of 0.897 should therefore be read as upper bounds derived from a selected, high-acuity subgroup rather than as the performance to be expected if APACHE-II were applied to every operated patient at admission; a score restricted to intensive-care patients also cannot serve the admission-triage purpose for which the wider panel is proposed.

One structural limit of the prognostic analysis follows directly from the design: because MPI and APACHE-II are operative and physiologic scores, they were neither applicable nor computed in the non-operative NSAP controls. The severity-score and mortality analyses are therefore confined to the operative cohort (AMI + NSII), and no prognostic comparison with NSAP is possible—the control group serves only to anchor the diagnostic biomarker comparison, not the outcome analysis.

### 4.4. Strengths and Limitations

The main strength is the three-arm design. Instead of comparing AMI with healthy or mixed controls, we set it against both a non-vascular ischemic comparator and a realistic non-specific abdominal pain group, so each marker could be judged against the two questions a surgeon actually asks. The analysis was specified per group, deposited externally, and validated internally by bootstrap resampling with calibration and decision-curve assessment; every figure reported here can be reproduced from the audited dataset and code.

The limitations are real. The study is retrospective and single-center, with the usual risks of selection and information bias and limited generalizability. Laboratory data were incomplete in the control group, where some tests were ordered selectively; the resulting non-random missingness inflates several diagnostic AUCs. Follow-up of the NSAP controls was limited to the index admission, so an early post-discharge misdiagnosis cannot be formally excluded, although none re-presented for operation during that admission. The mortality models were limited by the number of events. The 30-day model had only 5.8 events per variable and calibrated imperfectly (Hosmer–Lemeshow *p* = 0.009), so its coefficients are exploratory, and its optimism-corrected discrimination (0.69–0.73 after bootstrap) is the honest estimate. APACHE-II was scored only in the intensive-care subset (*n* = 76), so the combined-score analysis is confined to the sickest patients; this restriction is a selection bias that probably inflates the apparent discrimination of APACHE-II, and the APACHE-II and combined-model areas under the curve are accordingly best read as upper bounds obtained in a selected high-acuity subgroup rather than as cohort-wide estimates (Section 4.3). Finally, because calendar dates of death were not recorded, survival analysis stopped at the index admission; the 30- and 90-day figures coincide because every death within 90 days happened in hospital, and longer-term outcomes were beyond reach. Prospective, multicenter work with complete biomarker capture and external validation is needed before this admission panel can be recommended for routine triage.

## 5. Conclusions

In suspected acute intestinal ischemia, admission inflammatory and nutritional indices distinguished transmural ischemia from non-specific abdominal pain with high accuracy but did not reliably separate vascular from non-vascular causes, for which CT angiography remained essential. Within this cohort, in-hospital mortality was best predicted by the physiology-based severity scores APACHE-II and MPI, strengthened in combination, together with chronic kidney disease and arterial lactate. Rather than recommending any of these measures for immediate clinical adoption, we interpret them as internally consistent, hypothesis-generating observations from a single center. The panel described here should therefore be regarded as a hypothesis to be tested rather than as a validated triage instrument; it has not been externally validated, its internally derived performance estimates are optimistic—those for APACHE-II additionally originating from a selected intensive-care subset—and it must not be used to guide decisions in individual patients in its present form. Prospective, multicenter studies with complete biomarker capture and external validation are required to determine whether a composite admission panel can improve early triage and prognostication in this highly lethal condition.

## Figures and Tables

**Table 1:** Baseline demographic and comorbidity profile across the three diagnostic groups.

Characteristic	AMI (*n* = 61)	NSII (*n* = 62)	NSAP (*n* = 74)	*p* †
Age, years (mean ± SD)	63.9 ± 15.9	63.2 ± 17.4	56.0 ± 12.9	<0.001
Female sex	37 (60.7)	31 (50.0)	41 (55.4)	0.49
Coronary artery disease	14 (23.0)	11 (17.7)	20 (27.0)	0.44
Heart failure	12 (19.7)	7 (11.3)	2 (2.7)	0.006
Atrial fibrillation	19 (31.1)	9 (14.5)	8 (10.8)	0.006
Cerebrovascular disease	9 (14.8)	4 (6.5)	1 (1.4)	0.010
Prior thromboembolism	8 (13.1)	1 (1.6)	1 (1.4)	0.003
Dyslipidemia	5 (8.2)	0 (0.0)	7 (9.5)	0.051
Hypertension	34 (55.7)	29 (46.8)	37 (50.0)	0.60
Diabetes mellitus	20 (32.8)	12 (19.4)	28 (37.8)	0.059
Chronic kidney disease	22 (36.1)	9 (14.5)	0 (0.0)	<0.001
COPD/asthma	11 (18.0)	9 (14.5)	9 (12.2)	0.63
Hypercoagulable state	17 (27.9)	12 (19.4)	2 (2.7)	<0.001
Inflammatory bowel disease	2 (3.3)	1 (1.6)	1 (1.4)	0.70
Smoking	7 (11.5)	10 (16.1)	2 (2.7)	0.026
COVID-19	2 (3.3)	1 (1.6)	0 (0.0)	0.30

Data are *n* (%) unless otherwise stated. † *p* from the Kruskal–Wallis test (age) or the Pearson chi-square test (categorical variables), which was applied to every categorical row of this three-group comparison; the minimum expected cell count was below five in five rows (cerebrovascular disease, prior thromboembolism, dyslipidemia, inflammatory bowel disease, and COVID-19), and the corresponding *p* values should be read with that in mind. AMI, acute mesenteric ischemia; NSII, non-vascular (extravascular) intestinal ischemia; NSAP, non-specific abdominal pain; COPD, chronic obstructive pulmonary disease.

**Table 2:** Focused comparison of the two operative groups (AMI vs. NSII).

Characteristic	AMI (*n* = 61)	NSII (*n* = 62)	*p*
Age, years (mean ± SD)	63.9 ± 15.9	63.2 ± 17.4	0.94
Female sex	37 (60.7)	31 (50.0)	0.23
Coronary artery disease	14 (23.0)	11 (17.7)	0.47
Heart failure	12 (19.7)	7 (11.3)	0.20
Atrial fibrillation	19 (31.1)	9 (14.5)	0.028
Cerebrovascular disease	9 (14.8)	4 (6.5)	0.13
Prior thromboembolism	8 (13.1)	1 (1.6)	0.017
Dyslipidemia	5 (8.2)	0 (0.0)	0.028
Hypertension	34 (55.7)	29 (46.8)	0.32
Diabetes mellitus	20 (32.8)	12 (19.4)	0.090
Chronic kidney disease	22 (36.1)	9 (14.5)	0.006
COPD/asthma	11 (18.0)	9 (14.5)	0.60
Hypercoagulable state	17 (27.9)	12 (19.4)	0.27
Inflammatory bowel disease	2 (3.3)	1 (1.6)	0.62
Smoking	7 (11.5)	10 (16.1)	0.45
COVID-19	2 (3.3)	1 (1.6)	0.62

Data are *n* (%) unless otherwise stated. *p* from the Mann–Whitney U test (age) or the Pearson chi-square test, with the Fisher exact test where any expected cell count was below five (prior thromboembolism, dyslipidemia, inflammatory bowel disease, and COVID-19). This table corresponds to the original Table 1 of the submitted manuscript. ICU, intensive care unit.

**Table 3:** Admission inflammatory and prognostic biomarkers across the three diagnostic groups.

Biomarker (Mean ± SD)	AMI (*n* = 61)	NSII (*n* = 62)	NSAP (*n* = 74)	*q* *
WBC count (×10^9^/L)	16.3 ± 10.0	12.2 ± 6.6	7.9 ± 1.9	<0.001
Neutrophil-to-lymphocyte ratio	14.8 ± 11.1	10.0 ± 9.2	2.5 ± 2.2	<0.001
Platelet-to-lymphocyte ratio	319.8 ± 216.2	279.7 ± 256.0	129.6 ± 99.4	<0.001
Neutrophils (%)	82.8 ± 12.9	76.3 ± 17.3	58.6 ± 12.4	<0.001
Lymphocytes (%)	9.4 ± 8.5	11.2 ± 9.9	30.8 ± 11.5	<0.001
Red-cell distribution width (%)	15.6 ± 3.2	14.6 ± 2.2	13.6 ± 1.1	<0.001
Immature granulocytes (%)	1.67 ± 2.56	0.94 ± 1.42	0.29 ± 0.17	<0.001
Hemoglobin (g/dL)	11.8 ± 2.4	13.7 ± 4.1	13.4 ± 1.5	<0.001
Arterial lactate (mmol/L)	4.14 ± 3.62	2.43 ± 1.97	1.65 ± 1.00	<0.001
Albumin (g/L)	31.5 ± 8.2	36.7 ± 12.1	43.0 ± 3.8	<0.001
Creatinine (mg/dL)	1.99 ± 2.05	1.17 ± 0.75	0.79 ± 0.17	<0.001
Urea (mg/dL)	69.3 ± 40.9	55.9 ± 44.4	35.9 ± 33.0	<0.001
eGFR (mL/min/1.73 m^2^)	54.2 ± 33.3	73.5 ± 33.7	90.7 ± 23.1	<0.001

* Kruskal–Wallis test with Benjamini–Hochberg adjustment (*q* < 0.001 for all listed markers). Per-group denominators for markers with missing data (AMI/NSII/NSAP): arterial lactate 54/34/18, albumin 52/51/23, creatinine 61/61/71, urea 61/61/72, eGFR 46/50/54, immature granulocytes 28/31/34; all other listed markers were available in every patient (61/62/74). WBC, white blood cell; eGFR, estimated glomerular filtration rate.

**Table 4:** Diagnostic discrimination of admission markers (ROC analysis).

**Panel A. AMI vs. NSAP (Control)**						
**Marker**	**AUC (95% CI)**	**Cut-off**	**Sens (%)**	**Spec (%)**	**PPV (%)**	**NPV (%)**
Neutrophils (%)	0.932 (0.884–0.979)	>73.0	85.2	93.2	91.2	88.5
Neutrophil-to-lymphocyte ratio	0.921 (0.869–0.972)	>3.97	85.2	93.2	91.2	88.5
Immature granulocytes (%)	0.903 (0.822–0.984)	>0.35	92.9	79.4	78.8	93.1
Albumin	0.892 (0.820–0.963)	<40.54	80.8	87.0	93.3	66.7
Prognostic nutritional index	0.872 (0.792–0.952)	<45.19	73.1	95.7	97.4	61.1
Platelet-to-lymphocyte ratio	0.830 (0.753–0.907)	>213.81	67.2	93.2	89.1	77.5
RDW-SD	0.807 (0.689–0.925)	>46.4	65.5	94.1	90.5	76.2
Arterial lactate	0.781 (0.665–0.898)	>1.93	72.2	77.8	90.7	48.3
Creatinine	0.773 (0.684–0.862)	>0.97	65.6	90.1	85.1	75.3
**Panel B. AMI vs. NSII**						
**Marker**		**AUC (95% CI)**	**Cut-off**	**Sens (%)**		**Spec (%)**
Arterial lactate		0.672 (0.558–0.787)	>2.39	61.1		73.5
Neutrophils (%)		0.659 (0.562–0.756)	>88.1	47.5		82.3
Immature granulocytes (%)		0.650 (0.510–0.791)	>0.35	92.9		32.3
Neutrophil-to-lymphocyte ratio		0.643 (0.544–0.742)	>10.01	60.7		67.7
Albumin		0.628 (0.520–0.737)	<31.79	59.6		64.7
RDW-SD		0.627 (0.478–0.776)	>46.4	65.5		73.3

AUC, area under the receiver operating characteristic curve; PPV/NPV, positive/negative predictive value; cut-offs by the Youden index.

**Table 5:** Severity, operative, and outcome characteristics of the operative groups.

Characteristic	AMI (*n* = 61)	NSII (*n* = 62)	*p*
Mannheim Peritonitis Index (mean ± SD)	23.0 ± 9.3	17.5 ± 7.4	<0.001
Length of stay, days (median)	10	7	0.003
Small-bowel resection, cm (median)	87.5	30	<0.001
Organ failure	43 (70.5)	37 (59.7)	0.21
Preoperative sepsis > 24 h	39 (63.9)	20 (32.3)	<0.001
Malignancy	11 (18.0)	5 (8.1)	0.10
Non-colonic sepsis source	36 (59.0)	58 (93.5)	<0.001
Diffuse generalized peritonitis	13 (21.3)	3 (4.8)	0.007
Purulent intra-abdominal exudate	29 (47.5)	10 (16.1)	<0.001
Abdominal tenderness	49 (80.3)	32 (51.6)	<0.001
Guarding/rebound	18 (29.5)	4 (6.5)	<0.001
Postoperative ICU admission	53 (86.9)	30 (48.4)	<0.001
In-hospital mortality (primary endpoint)	29 (47.5)	10 (16.1)	<0.001
30-day mortality (secondary)	26 (42.6)	10 (16.1)	0.001
90-day mortality (secondary)	29 (47.5)	10 (16.1)	<0.001

Data are *n* (%) unless otherwise stated. ICU, intensive care unit.

**Table 6:** Single-marker and combined-model discrimination for mortality (operated patients).

**Panel A. Single-Marker AUC (95% CI)**			
**Marker**	**In-Hospital**	**30-Day**	**90-Day**
APACHE-II	0.861 (0.774–0.948)	0.855 (0.762–0.948)	0.861 (0.774–0.948)
Mannheim Peritonitis Index	0.810 (0.723–0.897)	0.792 (0.699–0.886)	0.810 (0.723–0.897)
Prognostic nutritional index	0.755 (0.654–0.855)	0.747 (0.643–0.850)	0.755 (0.654–0.855)
Arterial lactate	0.694 (0.581–0.807)	0.680 (0.564–0.796)	0.694 (0.581–0.807)
Neutrophil-to-lymphocyte ratio	0.664 (0.563–0.765)	0.638 (0.533–0.743)	0.664 (0.563–0.765)
**Panel B. Combined Severity Model (In-Hospital Death)**			
**Model**	**AUC (95% CI)**		**DeLong vs. MPI**
MPI alone	0.772 (0.663–0.882)		—
APACHE-II alone	0.861 (0.774–0.948)		0.241
MPI + APACHE-II (combined)	0.897 (0.829–0.964)		0.014

In-hospital and 90-day estimates coincide because all deaths within 90 days occurred during the index admission. APACHE-II was available in the ICU-scored subset (*n* = 76); Panel B is restricted to that subset, so its MPI AUC (0.772) is lower than in the full operative cohort of Panel A (0.810). The combined model improved on MPI alone (DeLong *p* = 0.014) but did not differ significantly from APACHE-II alone (DeLong *p* = 0.257).

**Table 7:** Multivariable logistic regression for 30-day and 90-day mortality (*n* = 88).

Predictor	30-Day aOR (95% CI)	*p*	90-Day aOR (95% CI)	*p*
Age (per year)	1.02 (0.98–1.06)	0.29	1.03 (0.99–1.07)	0.16
Mannheim Peritonitis Index (per point)	1.12 (1.05–1.21)	0.001	1.14 (1.06–1.23)	<0.001
Neutrophil-to-lymphocyte ratio	1.02 (0.97–1.07)	0.54	1.02 (0.97–1.08)	0.40
Arterial lactate (per mmol/L)	1.16 (1.00–1.38)	0.065	1.20 (1.03–1.45)	0.031
Atrial fibrillation	0.42 (0.12–1.33)	0.15	0.73 (0.22–2.34)	0.60
Chronic kidney disease	2.96 (1.02–9.14)	0.0496	3.72 (1.25–12.10)	0.022

aOR, adjusted odds ratio (Enter method; profile-likelihood CIs). Events: 35 (30-day) and 38 (90-day). Reported as exploratory owing to the limited events-per-variable ratio. Given the limited sample size and events-per-variable ratio, the adjusted odds ratios should be interpreted with caution and read as directional rather than precise effect estimates. The 30-day *p* value for chronic kidney disease is given to four decimal places because it lies immediately below the 0.05 threshold (exact value 0.0496).

## Data Availability

The analysis code, statistical outputs, figures, and Supplementary Material Tables are openly available on Zenodo (https://doi.org/10.5281/zenodo.20600497) [14]. The de-identified individual-patient dataset is available from the corresponding author upon reasonable request.

## Supplementary Materials

The following supporting information—the analysis code, the complete statistical outputs, the supplementary figures (biomarker ROC, internal-validation calibration, decision-curve, and Kaplan–Meier analyses), and the supplementary tables—can be downloaded at https://doi.org/10.5281/zenodo.20600497.
